# “You can push these conversations, but don’t push your patient away”: healthcare learner perspectives on virtual simulation games as an educational approach to address vaccine hesitancy

**DOI:** 10.3389/fpubh.2024.1408871

**Published:** 2024-07-03

**Authors:** Emily J. Doucette, Margaret Pateman, Madison M. Fullerton, Alyssa Lip, Sherilyn K. D. Houle, James D. Kellner, Jenine Leal, Shannon E. MacDonald, Deborah McNeil, Sandra Davidson, Cora Constantinescu

**Affiliations:** ^1^Department of Pediatrics, Cumming School of Medicine, University of Calgary, Calgary, AB, Canada; ^2^Faculty of Nursing, University of Calgary, Calgary, AB, Canada; ^3^19 to Zero Inc., Rocky Mountain House, AB, Canada; ^4^Division of Respirology, Department of Medicine, University of Calgary, Calgary, AB, Canada; ^5^School of Pharmacy, University of Waterloo, Waterloo, ON, Canada; ^6^Alberta Children’s Hospital Research Institute, University of Calgary, Calgary, AB, Canada; ^7^Department of Community Health Sciences, Cumming School of Medicine, University of Calgary, Calgary, AB, Canada; ^8^Department of Microbiology, Immunology and Infectious Diseases, University of Calgary, Calgary, AB, Canada; ^9^O’Brien Institute for Public Health, University of Calgary, Calgary, AB, Canada; ^10^Infection Prevention and Control, Alberta Health Services, Calgary, AB, Canada; ^11^Antimicrobial Resistance - One Health Consortium, University of Calgary, Calgary, AB, Canada; ^12^Real World Evidence Consortium, University of Calgary, Calgary, AB, Canada; ^13^Faculty of Nursing, University of Alberta, Edmonton, AB, Canada; ^14^Maternal Newborn Child and Youth Strategic Clinical Network, Alberta Health Services, Calgary, AB, Canada; ^15^Pediatric Infectious Diseases, Department of Pediatrics, Cumming School of Medicine, University of Calgary, Calgary, AB, Canada

**Keywords:** virtual simulation, vaccine hesitancy, communication, thematic analysis, focus groups, qualitative research

## Abstract

**Background:**

Vaccine hesitancy is a significant threat to public health. Healthcare providers (HCPs) can address hesitancy during routine patient conversations; however, few multidisciplinary education tools exist for HCPs to learn to engage in vaccine discussion especially considering new vaccine technologies such as mRNA vaccines. The objectives of this study were to explore HCP learners’ experiences with COVID-19 vaccine communication, and qualitatively evaluate an online learning module composed of virtual simulation games (VSGs) which utilize the PrOTCT Framework for HCP vaccine communication.

**Methods:**

Three virtual focus groups were conducted from December 2022 to January 2023 with Canadian healthcare learners in nursing (*N* = 6), pharmacy (*N* = 9), and medicine (*N* = 7) who participated in a larger study measuring the effectiveness of the VSGs. Using a pragmatic approach, a qualitative thematic analysis was conducted using NVivo to identify themes and subthemes.

**Results:**

A total of 22 HCP learners participated in this study and three key themes were identified. Across all three disciplines, participants expressed that (1) their prior education lacked training on how to hold vaccine conversations, resulting in uncomfortable personal experiences with patients; (2) the VSGs increased their confidence in holding vaccine conversations by providing novel tools and skills; and (3) participants also provided feedback to improve the VSGs which was implemented and supported the dissemination to all HCP professions.

**Conclusion:**

Although HCPs are a trusted source of vaccine information, participants in this study felt they received little training on how to engage in challenging conversations regarding COVID-19 vaccines. The introduction of the PrOTCT Framework and presumptive statements provided novel strategies for HCP to initiate vaccine conversations, especially considering new vaccine technologies and participants appreciated the emphasis on coping strategies and resilience. It is essential that HCP are provided both opportunities to practice managing these conversations, and tools and skills to succeed at an early point in their careers to prepare them for future roles in vaccine advocacy, delivery, and promotion.

## Introduction

Vaccine hesitancy is defined by the World Health Organization as the delay or refusal to accept vaccines despite availability (1). The emergence of widespread skepticism and mistrust of vaccines among the vaccine-hesitant population, as well as other barriers to immunization, has led to a dangerous global decrease in rates across several vaccination programs (2, 3). The number of Canadian parents who report being “really against” vaccinations for their children has increased dramatically from 4% in 2019 to 17% in 2024 (4). This increase in hesitancy was exacerbated as a result of the COVID-19 pandemic and the rapid development of mRNA vaccines against SARS-CoV-2 (5, 6) resulting in vaccine hesitancy remaining one of the top critical threats to global health and well-being (7). Even those who were historically accepting of other vaccines reported increased concerns regarding mRNA COVID-19 vaccines, as a result of the novelty of the vaccines, changing public health guidelines and recommendations, and online misinformation (5, 8).

Following the pandemic, healthcare providers (HCPs) have reported feeling uncomfortable initiating vaccine discussions for several reasons, including but not limited to time constraints, staffing limitations, competing priorities, individual burnout, lack of organizational support, and the overall erosion of trust in the healthcare system (9–14). As a result, urgent action is needed to develop effective strategies for HCP to use to combat vaccine hesitancy and encourage the acceptance of vaccines (6, 15). Vaccine discussion is a complicated interpersonal interaction that requires verbal and non-verbal communication skills to actively listen, recognize hesitancy, and address denialism, as pro-vaccine messaging alone is unlikely to be effective (16–19). Whereas initially treated as a knowledge deficit phenomenon, more recent work recognizes vaccine hesitancy as a complex by-product of a person’s lived and collective experiences with illness, biomedical institutions, injustices, and their relationships with government and the scientific community (20–22). Rather than viewing these individuals as one homogenous category, HCPs need both knowledge and communication tools to respond in a tailored fashion to individual vaccine hesitant archetypes (23) to effectively engage, build trust, and advance vaccination intention.

A recent scoping review conducted by Lip et al. (24) examined whether educational interventions existed for HCPs on how to effectively engage in vaccine discussions. Several gaps were identified, such as limited accessibility of the interventions. Notably, the interventions were more targeted toward medical workers (students, residents, physicians, and physician assistants) and less so to other disciplines such as nursing and pharmacy, despite these providers playing an important role in immunization distribution and uptake. Similarly, a survey of American HCP students identified a lack of knowledge and overall discomfort engaging in vaccine discussion among medical, nursing, pharmacy students (25).

In response to this gap, we developed an online learning module consisting of three virtual simulation games (VSGs) to specifically address the need for more accessible interventions targeting HCPs across disciplines of medicine, nursing, and pharmacy (26, 27). A discipline and knowledge agnostic design was selected for the games to ensure the intervention could be completed regardless of practice setting or vaccine-specific knowledge but also to encourage future vaccine conversations and recommendations across disciplines. Each VSG was objective-based and designed to increase HCPs confidence and self-efficacy in vaccine communication through the use of presumptive statements (17) and the PrOTCT Framework (28), which are effective evidence-based tools developed to help HCPs discuss vaccines with patients. The PrOTCT Framework, based on evidence based principles of presumptive statements as well as motivational interviewing strategies (**P**resume the patient will vaccinate, **O**ffer to share knowledge and personal experiences with vaccines once you have explored their stance using OARS-open questions, affirmation, reflective listening and summarizing reflections, **T**ailor recommendations to address patients’ specific health **C**oncerns, and **T**alk through a specific plan for when and where to get vaccinated), was designed by experts and provides HCPs a framework for vaccine communication (28).

VSG1 focused on conversing with patients expressing hesitancy around receiving an mRNA vaccine booster or completing a vaccine series. VSG2 focused on conversing with patients who minimize the risk of diseases such as COVID-19 while maximizing the risk of the vaccine, especially newer mRNA vaccines. VSG3 focused on fostering HCPs’ personal resilience, building and maintaining self-efficacy, and provided suggestions to prevent burnout and moral distress when dealing with vaccine refusal. We chose to conduct a pilot evaluation of the VSGs with HCP learners in nursing, pharmacy, and medicine in an effort to provide learners with effective vaccine communication skills early in their careers. The objectives of this qualitative study were to: (1) explore HCP learners’ personal experiences with vaccine education and vaccine discussion, and (2) conduct a qualitative evaluation of the VSGs to identify opportunities for improvement prior to accreditation and dissemination of the VSGs.

## Methods

### Study design

This was a qualitative evaluation within a larger pilot study in which we conducted three focus groups between December 2022 and January 2023 with students in nursing from the University of Calgary, students in pharmacy from the University of Waterloo, and medical residents in the internal medicine, family medicine, public health, pediatrics, obstetrics and gynecology, and emergency medicine specialties from the University of Calgary who provided informed consent. Focus groups were used to foster valuable discussions to identify the opinions of and recommendations for the VSGs. This study received approval from the University of Calgary Conjoint Health Research Ethics Board (REB22-0012) and a University of Waterloo Research Ethics Board (REB 44487).

### Participant recruitment

Participants were recruited for the focus groups from an existing cohort of 72 participants who had completed all 3 VSGs as part of a larger project evaluating the effectiveness of the VSGs at improving learners’ confidence in addressing vaccine hesitancy (27). Eligibility was defined as medical residents in the specialties of Internal Medicine (IM), Family Medicine (FM), Obstetrics and Gynecology (OBGYN), pediatrics (Peds), Emergency Medicine (EM), nursing students in the third or fourth year of their program, and pharmacy students in second, third, or fourth year of their program, as they were most likely to have previous clinical experiences discussing vaccines. Participants were offered an electronic gift card for their time spent participating in the focus group for CAD $50.

### Focus group guide development

The focus group guide was developed by the project team based on the findings from the scoping review (24) that outlined the gaps in vaccine conversation education. Questions were designed to explore participants’ past experiences with vaccine communication, prior education they received on vaccine communication, as well as their experiences with the VSGs specifically exploring the user experience and their perceived confidence with vaccine discussions.

### Focus group moderation/data collection

Discipline-specific focus groups were led by two female members of the research team who were approximately the same age as the participants and were experienced in qualitative methodology. The focus groups were conducted online using Zoom (Zoom Video Communications, Inc., San Jose, CA). Focus groups were 1–1.5 h in length and were led by one researcher, while one facilitator observed, took field notes, and provided technical support. Following the focus groups, the moderator and facilitator debriefed and shared field notes with each other.

### Qualitative analysis

The focus groups were audio and video recorded, with third-party verbatim transcription to support rigorous data analysis. Two qualitative researchers performed iterative thematic analysis on transcribed data to identify key themes using Braun and Clarke’s 6 step framework (familiarize oneself with the data; generate initial codes; search for themes; review themes; define and name themes; produce final report) (29). Analysis was conducted until thematic saturation was reached to support the dependability of our findings. Data organization and analysis were conducted using the qualitative data analysis software, NVivo 12 (30). The coding and thematic analysis was supported by reviewing field notes recorded during each focus group and comparing the emergent findings to ensure no key themes were missed. Regular communication between the researchers ensured that potential biases from their subjective experiences were addressed and changes to the analysis were discussed and agreed upon. In the discussion of themes, quotations from participants are provided along with the participants’ discipline and year of program.

## Findings

Of the 72 potential participants from the larger pilot study, 22 participated in one of three discipline-specific focus groups; the distribution included 6 nursing students, 9 pharmacy students, and 7 medical residents. Overall, participants were predominantly female (95.4%). Twenty participants (90.9%) reported having a vaccine conversation in the past, while only 13 (59.1%) reported learning about how to have vaccine conversations in their program. There were no differences in the self-evaluation scores between those who participated in the focus groups and those who only participated in the larger pilot study, suggested the focus group sample adequately represented the larger study population. Additional participant characteristics are provided in Table 1. Three broad themes consistent across all three disciplines were identified through thematic analysis. Additional quotations and subthemes are included in Table 2.

**Table 1:** Focus group participant demographics.

	Nursing (*N* = 6)	Pharmacy (*N* = 9)	Medicine (*N* = 7)
Age, n (%)			
18–25	4 (66.7)	9 (100.0)	1 (14.3)
26+	2 (33.3)	0 (0.0)	6 (85.7)
Gender, n (%)			
Female	6 (100.0)	9 (100.0)	6 (85.7)
Male	0 (0.0)	0 (0.0)	1 (14.3)
Year of HCP program, n (%)			
1st	0 (0.0)	0 (0.0)	4 (57.1)
2nd	0 (0.0)	1 (11.1)	3 (42.9)
3rd	1 (16.7)	5 (55.6)	0 (0.0)
4th	5 (83.3)	3 (33.3)	0 (0.0)
Medical resident specialty, n (%)			
Pediatrics			2 (28.6)
Public Health and Preventative Medicine (PHPM)			1 (14.3)
Family Medicine			4 (57.1)

**Table 2:** Qualitative focus group themes, subthemes, and representative quotes from each HCP learner discipline.

Theme	
Subtheme	Key Quotes
**Theme 1: HCP learners’ prior education lacked practical training on how to have difficult conversations with patients, resulting in uncomfortable personal experiences discussing vaccines.**	
Participants prior education was very didactic and lacked training on soft communication skills and presumptive statements.	*“I honestly do not think we had very much training as far as having those conversations with patients. Like if I think back, way back to my term three, I think maybe there was some, like a little bit of training just as far as like vaccines just like on their own, but not necessarily like the conversations behind them as far as like, this is how you should approach it with a patient and XYZ”* (4^th^ year nursing student) *“The vaccine class, or the whole vaccine program that we did in school just felt more theoretical. It was mainly based on knowledge of vaccines. I do understand that we had other courses which touched upon patient communication and how to use active listening, like those soft skills, but there’s really no course that combines the two.”* (3^rd^ year pharmacy student) *“We did have just one lecture, I think, that addressed the vaccine hesitancy. And they did kinda give us some of the main reasons that people provide for being hesitant to vaccines and kinda some of the evidence to rebuttal that…”* (1^st^ year family medicine resident)
Participants felt uncomfortable and unprepared to have challenging vaccine conversations with patients, fearing it would hinder the therapeutic relationship.	*“I think when faced with a more difficult individual that’s like very passionate about their opposing beliefs or that’s just very strong-willed it makes me, I feel like I might get a little nervous ‘cause some individuals might get aggressive. I do not know if that’s an extreme, but I just know that some people are very passionate about their opinions in specific situations, and I tend to be a more non-confrontational individual in general. So just because of that I tend to be a little bit more hesitant if it was to take that negative, um, turning point.”* (4^th^ year nursing student) *“I was being pretty passive in [a] conversation, um, just because I did not really know how to respond and I was trying to provide reasons to that patient, just general reasons, like that everyone should get it, um, you never know. You cannot really protect against it. But I felt as though I could have been more active, but I did not know how I could direct the conversation and better convince them instead of just giving, you know, general things that they probably heard elsewhere.”* (3rd year pharmacy student) *“If you open up the record and see like, they have had no vaccines. Then all of a sudden, I’m like kind of a bit nervous (laughs) and thinking like, “Oh, gosh. How do I even have this conversation?”* (2^nd^ year family medicine resident)
Participants were motivated to complete the VSGs due to the importance of vaccine communication skills in their daily practice.	*“I think knowing that this information could be helpful for so many difficult clinical situations we might encounter, just knowing our profession is like what really kind of motivated me to want to do the games you could say … so just establishing that connection and understanding how common it is to see hesitancy, see or just face difficult situations or conversations with patients”* (4^th^ year nursing student) *“For me, working in a community pharmacy, I came across many vaccine hesitant patients and I did not know exactly how to deal with them, so I wanted to better my own skills and be more confident in that area, so that’s what motivated me a lot.”* (4^th^ year pharmacy student) *“I think just knowing that this is something that’s gonna come up over and over again in residency and in practice, and just wanting those skills, and recognizing that I do not have them or need some help”* (2^nd^ year pediatrics resident)
**Theme 2: HCP learners felt the educational intervention increased their confidence and self-efficacy in having challenging vaccine conversations by providing useful tools and new and transferable skills.**	
The emphasis on HCP resilience and coping strategies present in VSG 3 brought a unique and often overlooked approach for HCPs dealing with challenging conversations.	*“But I also like the part of the games where they are like, “Oh, like when you have difficult conversations with patients, you also have to have compassion for yourself.” I really like that part because I feel like we skip over that a lot. But like, going through the games kind of helped me recircle back to that point that you have to be kind with yourself even while having these conversations with patients.”* (4^th^ year nursing student) *“…for that last module, there was no real resolution, it did not have a happy ending of the patient ending up with a vaccine, and I think if that happened in real life, I would blame myself. I would say, “If it were someone else who were better at this than me, the patient would’ve ended up with the vaccine.” And I think that made me realize like this is a professional handling it, and it did not go the way that I wanted it to go, or the professional wanted it to go, so maybe it’s not my fault that this is happening, maybe the patient just was not ready for it today.”* (4th year pharmacy student) *“I’ll take away the self-compassion piece and the piece about, you know, you can push these conversations, but do not push your patient away. And knowing when to kind of take that step back to preserve the therapeutic relationship.”* (1st year PHPM resident)
Use of novel PrOTCT framework and presumptive statements	*“So I would say that definitely the presumptive language that the module introduced was something that was also quite surprising to me ‘cause it’s definitely not an approach that I had thought about before. I think initially when I had seen that in the modules, I actually thought it was quite an abrupt way to ask patients about their vaccines. It was something that I have not had much practice in the past before. And I’d say in general, the modules were a really good starting point to develop an approach to having these conversations.”* (2^nd^ years pediatrics resident) *“I had also never heard of it before and I had never used it before. And I did not really consider it until I saw it being used, like implemented in the videos. And I was, at first I was taken aback ‘cause I thought that you were not supposed to kind of assume, um, but to see how it’s laid out and how it’s used, um, I think it makes sense more now to me. And I kind of like... I appreciated having the modules because of that, because if I had never done it, then I would’ve never known and I would’ve continued on with my mindset of like, “Do not assume” and “do not, you know, do not go in that specific way.” So I really, really, appreciated that.”* (4^th^ year nursing student)
The VSGs content was discipline agnostic, making it multidisciplinary and applicable to many different patient scenarios (other vaccines, medications).	*“I think [the VSGs are] applicable to general vaccines and administration, and also medication hesitancy because both of those things are concepts that happen a lot… with people not knowing what it does or like having bad experiences in the past.”* (4th year nursing student) *“Two examples I can think of, outside of vaccines, that I can see myself using the skills that I learned from these modules include like for diabetes, patient starting insulin, and even recommending not pharmacologic changes to patient for lifestyle modifications, like smoking cessation. Um, just being personable, kind of like building that rapport with them was something that I took away from the module that we can really apply to any patient scenario, just to, you know, get the ball rolling.”* (3^rd^ year pharmacy student) *“I would definitely recommend them to … most, or any other health profession who’s seeing the public in a preventative health kind of way. I think that would apply to a lot of different disciplines, even if they are not set up to specifically provide vaccines or discuss them all the time. I think just having that background and being able to navigate some of those conversations that are inevitably going to come up, regardless of the healthcare setting, I think it would be... I think the skills are very transferable amongst health professions.”* (1^st^ year family medicine resident).
**Theme 3: HCP learners enjoyed the learning modules and provided actionable feedback on the content, suggestions for future games, and endorsed accreditation.**	
The VSGs were enjoyable, interactive, patient-oriented, and engaging through real-life scenarios and first-person perspectives.	*“I really like them. I found them very user friendly and there were aspects where I found myself a bit challenged. Overall it was pretty easy to navigate just through common sense. But, there were definitely areas where I would click the wrong response and then through the, explanation, I really appreciated the explanations that were provided for those wrong responses as they allowed me to kinda reflect on my way of thinking.* (4^th^ year nursing student) *“I really liked how in that last simulation game, the questions were embedded within, so you really felt like you were the healthcare provider providing the information to that patient… And I liked how you could see, okay, if I chose this option, let us see what happens and this why it’s like not the right option, and then it … with the correct answer, it walks you through it, and then you see how it plays out.”* (4th year pharmacy student) *“I enjoyed them actually. I thought the approaches discussed were... Like they were new to me. ‘Cause like I had mentioned earlier, I had just not had really any significant formal teaching around it. And I think what was helpful was the videos and then the questions afterwards … I might select X, Y, Z answer, and it turned out to be wrong, um, for a lot of them. But, then watching the videos around it was quite helpful. So honestly, I think it was a pretty good curriculum. I wish it was introduced into medical school earlier for us.”* (2^nd^ year family medicine resident)
Knowledge agnostic content complemented existing theoretical knowledge, but learners want additional information to integrate both	*“I kind of have mixed feelings about it. Um, positive and negative. Like positive in the fact that like, it was strengthening the knowledge that we already had about having those conversations. But like, um, I feel like it was really wonderful to get to know more of that stuff. But like, I also thought, like I would’ve also appreciated a little bit of a knowledge base. I do not know if that was the point of the games. But like, um, I would’ve also have appreciated maybe just a little bit of like how to integrate, like talking about the theory part of it with the patient as well”* (4th year nursing student) *“I do not think these videos need to cater toward the theoretical knowledge, they do a great way of working on soft skills in an online platform, and I think that’s something that’s, we do not really get much in school outside of the clinical labs. So these videos are a really great way of learning how to practice on those soft skills without having to do it in person.”* (3^rd^ year pharmacy student) *“I think these conversations are really balancing like the art and science of medicine. I think these modules are really good at giving an approach for developing those communication skills for having these difficult conversations with families. But I do think that since a lot of them are coming up with specific facts that they read online, that it is really important to know the facts, and the science, and the evidence behind their specific questions. And I think as a healthcare provider, I think it also can diminish their trust in us, and if we do not like to have the specific details and the evidence behind like their particular question that they are asking us. And so, I think that it’s important for us to be able to know, like some of the evidence and the research behind, like some of their questions that they have, uh, with regards to vaccines.”* (1st year pediatrics resident)
Accreditation of the VSGs by a governing body/integration into coursework will make the intervention more likely to be completed by more HCPs.	*“If this was like added on into [coursework] in some way ... like the school making us do it. I think like if you ask some students to do it themselves, they might not, but if you throw it into a course and it’s enforced ... like they will not see how the benefits play out until they actually do it.”* (4th year pharmacy student) *“I think [accreditation] would be nice too, because I’m in the process of interviewing for jobs right now. Um, and something that I’ve noticed that a lot of people ask is what, like external education, are you doing on top of school. Um, and so I’ve been bringing this one up.”* (4th year nursing student)

### Theme 1: HCP learners’ prior education lacked practical training on how to have difficult conversations with patients, resulting in uncomfortable personal experiences discussing vaccines

When asked about their prior education about vaccine conversations, participants in all three disciplines reported that their experiences were often didactic and lacked training on integrating communication skills with immunization content. The academic training often included information on how to administer vaccines and address common questions regarding vaccine ingredients and side effects, but rarely explored how to navigate challenging conversations.

*“The vaccine class, or the whole vaccine program that we did in school just felt more theoretical. It was mainly based on knowledge of vaccines. I do understand that we had other courses which touched upon patient communication and how to use active listening, like those soft skills, but there’s really no course that combines the two.”* (3rd year pharmacy student)

In addition, participants in all three disciplines frequently reported feeling nervous, uncomfortable, and unprepared to engage in challenging vaccine conversations with patients. These emotions often resulted in them responding passively or dismissively when patients brought up concerns, while others hoped to avoid the conversations entirely by not bringing up the topic of vaccines. Residents expressed their hesitancy to address the topic, because they feared that it would hinder the therapeutic relationship with the patient and lead to burnout or moral distress.

*“If I'm being totally honest, I've had a lot of negative experiences, so I'm kind of less and less motivated to really push vaccines on patients, which sounds kind of terrible as a new graduate now, … Like how much do I wanna burn myself out trying to sort of almost convince people?”* (2nd year family medicine resident)

Participants from all disciplines felt motivated to complete the VSGs as they recognized their lack of relevant education and self-confidence. They emphasized their desire to become more confident in their vaccine communication skills, as they felt they would be utilized often in their future roles as HCPs.

*“I think just knowing that this is something that's gonna come up over and over again in residency and in practice, and just wanting those skills, and recognizing that I don't have them or need some help.”* (2nd year pediatrics resident)

### Theme 2: HCP learners felt the educational intervention increased their confidence and self-efficacy in having challenging vaccine conversations by providing useful tools and novel and transferable skills

Participants in all three disciplines reported finding the VSGs content to be discipline agnostic, with emphasis on the “soft skills” for communication. They felt the VSGs would be a useful educational tool for all HCPs to complete as they provide skills that are applicable to many different patient scenarios (including other vaccines, medication counseling, and nonpharmacologic lifestyle changes). In addition, they felt that widespread training on vaccine conversations would be beneficial as patients may be more comfortable sharing information with certain providers over others.

*“I think it can be applicable to many other healthcare professionals as well, because at the end of the day, it really depends on who the patients are most comfortable sharing information with. So it might not be their pharmacist. It might not be their nurse. They may be more comfortable with, you know, their doctor or like another social worker... Depends on who they have that really good rapport with, so if that's another healthcare provider that's not in pharmacy or nursing, then they'll still benefit from this module.”* (4th year pharmacy student)

The content in the third VSG specifically focused on HCP resilience, coping strategies, and avoiding moral injury when difficult conversations do not go the way the HCP had planned. Learners in all disciplines appreciated the reminder about the importance of self-compassion and felt the VSG content was a unique and often overlooked strategy for HCPs to utilize when dealing with challenging patient conversations.

*“I’ll take away the self-compassion piece and the piece about, you know, you can push these conversations, but do not push your patient away. And knowing when to kind of take that step back to preserve the therapeutic relationship.”* (1st year public health resident)

Although participants had not learned about presumptive statements and the PrOTCT Framework before, they were enthusiastic about practicing the techniques and incorporating them into conversations with future patients. Participants in both nursing and medicine expressed their surprise regarding the effectiveness of presumptive statements, as they had been taught not to make assumptions about patients.

*“So I would say that definitely the presumptive language that the module introduced was something that was also quite surprising to me 'cause it's definitely not an approach that I had thought about before. I think initially when I had seen that in the modules, I actually thought it was quite an abrupt way to ask patients about their vaccines. It was something that I haven't had much practice in the past before. And I'd say in general, the modules were a really good starting point to develop an approach to having these conversations.”* (2nd year pediatrics resident)

### Theme 3: HCP learners enjoyed the learning modules and provided actionable feedback on the content, suggestions for future games, and endorsed accreditation

All participants found the VSGs to be enjoyable, interactive, and engaging due to the use of real-life patient scenarios and first-person filming perspectives. Participants also appreciated the opportunity to learn from wrong answers as well as correct ones; the VSGs provided learners the opportunity to see how a situation would change when wrong responses were selected, and why it was not the best option at that time.

*“I just think it was really well-formatted in the way that it was very interactive because I think sometimes... When we are taught therapeutic communication, you can read an entire textbook about it, but until you actually have that opportunity to do it, like in a case study type of situation, or even in like real-life experiences… That’s when you really start to understand the types of comebacks people might give to you, um, which makes it a lot more challenging.”* (4th year nursing student)

Participants in all three disciplines appreciated the unique knowledge agnostic VSG design that did not center around factual learning. They felt that the content complemented their existing theoretical knowledge, however all students expressed a desire for additional information regarding how to best integrate vaccine-specific knowledge with communication skills.

*“I think these conversations are really balancing like the art and science of medicine. I think these modules are really good at giving an approach for developing those communication skills for having these difficult conversations with families. But I do think that since a lot of them are coming up with specific facts that they read online, that it is really important to know the facts, and the science, and the evidence behind their specific questions.”* (1st year pediatrics resident)

Finally, participants provided feedback and suggestions to improve the VSGs immediately and for future games (Table 3). All participants supported accreditation of the VSGs by a governing body and the integration of the VSGs into healthcare training program curricula to make the intervention more likely to be completed by a larger number of HCPs.

**Table 3:** Suggested improvements for VSGs design and topics of interest for future games.

Suggested improvements for VSGs	Table of contents/back button to allow for movement within VSGs Open text responses to provide opportunity to come up with potential answers Video responses to provide participants an opportunity to practice saying their responses out loud More information on the PrOTCT Framework
Suggested topics for future games	“Selective hesitancy,” patients who prefer a certain vaccine brand over another (e.g., Pfizer vs. Moderna) Vaccine hesitancy in diverse patient populations (e.g., pediatrics, pregnancy, seniors) How to answer questions about vaccine ingredients (pharmacy specific)? Vaccine-specific games, e.g., (HPV, flu, shingles) (resident specific)

## Discussion

Focus groups with learners in medicine, nursing, and pharmacy were conducted to qualitatively evaluate three VSGs, as well as elicit narrative experiences of HCP learners in holding these conversations. Thematic analysis of focus groups transcripts resulted in the identification of three key themes. Overall, HCP learners in medicine, nursing, and pharmacy reported a lack of sufficient training on how to engage in challenging vaccine conversations. However, they felt that the online learning module complemented their prior education on immunizations and increased their confidence holding these conversations by providing novel tools and useful skills. The VSGs were perceived to be a positive and useful learning modality for broader distribution. Our online learning module has the potential to address some of the current gaps in HCP knowledge and education.

Participants from all three disciplines felt the education they received was didactic, generalized, and did not provide training on how to integrate soft communication skills or presumptive statements into vaccine discussions. This is supported by a previous assessment of medical, nursing, and pharmacy school immunization curricula which found that curriculum content focused on immunization practices and principles rather than communication skills (31). Further, the time spent on the topic varied significantly by discipline and school, lacking consistency even within disciplines (31). Concerningly, HCP learners felt uncomfortable or unprepared when vaccine conversations arose, possibly related to the lack of training. It has been shown that the overall preparedness of a HCP is an important factor in their willingness to engage in conversations with patients (32), however even practicing HCP have expressed discomfort when the topic of vaccines is brought up by patients as a result of the pandemic (9–11). Participants recognized their lack of confidence, the importance of these conversations, and the frequency with which they will occur in practice, which was a motivating factor in completing the learning modules.

The VSGs resulted in an increase in participants’ reported willingness to engage in vaccine conversations with patients across all three disciplines. The use of gamification and virtual simulation in medical education is increasing in popularity as it has been shown to increase learner interest, motivation, and overall engagement, while reducing fear of failure (33, 34). Highlights of the VSGs were the virtual and gamified format, as participants reported they were engaging, interactive, multidisciplinary, and provided transferable skills, meaning they could be used by any HCP engaging in conversations with patients, both vaccine-related or otherwise. Considering the effect COVID-19 vaccines and mRNA technology mistrust has on public perception of vaccination, enabling HCP with transferable communication tools may positively contribute to trust building in the HCP-patient relationship (4, 8). The introduction of the evidence-based PrOTCT Framework (28) for guiding vaccine conversations was a novel technique across all three disciplines. Nursing and medical learners found presumptive statements contradicted their prior education about avoiding making assumptions about patients. This suggests a need for HCP training programs to integrate the use of presumptive statements in their immunization education, as presumptive statements have been shown to be significantly more effective than participatory statements in decreasing the odds of parental resistance to vaccines (17). All three disciplines found the content of the VSGs useful, supporting the use of the learning module as a multidisciplinary educational tool.

A unique aspect of the VSGs identified and celebrated by all three disciplines was the emphasis on HCP resilience, coping strategies, and strategies to preserve the therapeutic relationship. Participants enjoyed the reminder of the importance of self-compassion in healthcare professions, especially during difficult or adversarial conversations with patients. To our knowledge, no other vaccine communication interventions specifically address the management of HCP emotions (24), despite findings that the emotional state of HCPs significantly impacts their ability to have challenging conversations (35–37). Moral distress is the psychological distress experienced by HCP as a result of morally challenging situations (38–40), such as in instances of vaccine refusal, and has been associated with HCPs leaving their professions. While research exists to measure and address moral distress, including strategies such as specialist consultations, reflective debriefing, and educational interventions, further rigorous research is needed in this area (41). As HCP are at an increased risk of burnout, anxiety, and depression now more than ever, it is essential for institutions to not only provide mental health resources following burnout, but to provide strategies and training to avoid and address moral distress early in HCP education (37, 42, 43).

Participants supported accreditation and inclusion of the modules in HCP training programs to increase awareness and use of the VSGs, which will be facilitated alongside open access to the learning modules. We also explored potential areas for improvement. Participants appreciated that the learning modules did not require any specific knowledge of vaccines, but requested additional information about responding to specific vaccine questions which was not included in the games. This provides an opportunity for future VSGs to be added alongside the presently created ones, and for the VSGs to be incorporated into a single resource alongside other useful information for further education.

## Limitations

The study has several limitations. First, only three focus groups were conducted as this was part of a pilot evaluation of the VSGs, although thematic saturation was met after completion of the three focus groups. While there is some debate regarding the number of focus groups required, previous work on qualitative methodology has identified that over 80% of all themes are discovered within two to three focus groups (44). Further, the purpose of our study was quite narrow and specifically designed to identify participant experiences with the VSGs rather than understand deeper issues in medical education and vaccinology (45). Ultimately, further research is needed to confirm our findings. Recruitment of HCP learners in each discipline in the larger pilot study was also challenging due to HCP training programs’ rigorous academic demands, which resulted in a limited convenience sample of participants. To mitigate the impact, we offered multiple date and time options to participants to increase attendance. Medicine and pharmacy learners in this sample identified as female at a much higher rate than in the Canadian HCP population (46), although the percentage of female nursing learners in this sample was similar to the percentage of female nurses in Canada (47). Further, selection bias likely occurred due to our recruitment strategy and therefore we may have unknowingly missed learners with very high and low levels of self-confidence as they may have been less likely to enroll in a study on improving confidence due to fear of embarrassment or indifference. Lastly, it is important to acknowledge the role of both qualitative researchers and that their personal experiences, assumptions, and beliefs may have influenced the thematic analysis and what they deemed to be key themes.

## Conclusion

This qualitative evaluation adds to the growing literature emphasizing the important role HCPs play as a trusted source of vaccine information (32, 48–50), and the effectiveness of VSGs as an additional educational tool for HCP training (51). Our findings suggest that the VSGs have the potential to effectively address the need for a discipline and knowledge agnostic educational tool to increase the confidence of Canadian HCP learners, however further research with a larger number of participants is needed to both confirm and improve the reliability of our findings. The VSGs improved participants confidence by introducing new skills, such as the use of presumptive statements, and through a focus on HCP resiliency that can complement existing immunization and communication training. Ultimately, it is essential that HCP gain exposure to challenging vaccine conversations at an early point in their training to prepare them for their futures involving of mRNA vaccine advocacy, delivery, and promotion.

## Data availability statement

The raw data supporting the conclusions of this article will be made available by the authors, without undue reservation.

## Ethics statement

The studies involving humans were approved by the University of Calgary Conjoint Health Research Ethics Board (REB22-0012) and a University of Waterloo Research Ethics Board (REB 44487). The studies were conducted in accordance with the local legislation and institutional requirements. The participants provided their written informed consent to participate in this study.

## Author contributions

ED: Data curation, Formal analysis, Investigation, Methodology, Project administration, Validation, Writing – original draft, Writing – review & editing, Conceptualization. MP: Conceptualization, Data curation, Formal analysis, Project administration, Writing – original draft, Writing – review & editing, Investigation, Methodology. MF: Conceptualization, Methodology, Project administration, Writing – review & editing, Investigation. AL: Conceptualization, Investigation, Methodology, Writing – review & editing, Supervision. SH: Conceptualization, Investigation, Methodology, Supervision, Writing – review & editing. JK: Conceptualization, Methodology, Supervision, Writing – review & editing, Investigation. JL: Conceptualization, Methodology, Supervision, Writing – review & editing, Investigation. SM: Conceptualization, Methodology, Supervision, Writing – review & editing, Investigation. DM: Conceptualization, Methodology, Supervision, Writing – review & editing, Investigation. SD: Conceptualization, Funding acquisition, Methodology, Project administration, Supervision, Writing – review & editing, Investigation. CC: Writing – review & editing, Conceptualization, Funding acquisition, Investigation, Methodology, Project administration, Supervision.
